# Gendered Pathways Toward STEM Careers: The Incremental Roles of Work Value Profiles Above Academic Task Values

**DOI:** 10.3389/fpsyg.2018.01111

**Published:** 2018-07-02

**Authors:** Jiesi Guo, Jacquelynne Sue Eccles, Florencia M. Sortheix, Katariina Salmela-Aro

**Affiliations:** ^1^Institute for Positive Psychology and Education, Australian Catholic University, Sydney, NSW, Australia; ^2^School of Education, University of California, Irvine, Irvine, CA, United States; ^3^Department of Education, University of Helsinki, Helsinki, Finland; ^4^Department of Psychology, University of Jyväskylä, Jyväskylä, Finland

**Keywords:** gender differences, work values, task values, STEM, career choice

## Abstract

Drawing on Eccles' expectancy-value model of achievement-related choices, we examined how work values predict individual and gender differences in sciences, technology, engineering, and math (STEM) participations in early adulthood (ages of 25/27, 6 or 8 years after postsecondary school), controlling for subjective task values attached to academic subjects in late adolescence (11th grade, age 18). The study examined 1,259 Finnish participants using a person-oriented approach. Results showed that: (a) we could identify four profile groups based on five core work values (society, family, monetary, career prospects, and working with people); (b) work-value profiles predicted young adults actual STEM participation in two fields: math-intensive and life science occupations above and beyond academic task values (e.g., math/science) and background information; (c) work-value profiles also differentiate between those who entered support- vs. professional-level STEM jobs; and (d) gender differences in work value profiles partially explained the differential representation of women across STEM sub-disciplines and the overall underrepresentation of women in STEM fields.

## Introduction

Like the labor market and optional educational courses in general, women and men are differentially represented across the various science, technology, engineering, and math (STEM) fields (Valla and Ceci, 2014). For example, women are underrepresented in math-intensive fields of STEM education, such as mathematics, physical science, engineering, and computer science (hereafter math-intensive) but overrepresented in health, biological, and medical sciences (hereafter life science, OECD, 2014, 2016). Further, on average across OECD countries, 15-year-old girls are almost three times more likely as boys to aspire a career in a life science field, with the reverse being true regarding gender differences in career aspirations in math-intensive fields (OECD, 2016). The gender disparities are also apparent with tertiary degree enrolments, where women accounted for 78% of total enrolments in life science courses, but only 30% of total enrolments in science and engineering courses (OECD, 2014, also see Wang and Degol, 2017). Eccles' expectancy-value theory has been widely used to explain individual and gender differences in educational and career choices (Eccles, 2009). During adolescence, one's subjective task values (i.e., enjoyment, importance, usefulness, and negative cost) placed on different school subjects are assumed to influence academic and career pathways more so than one's history of academic performance. Academic task value in a domain has been found to be positively linked to knowledge acquisition and aspirations in said domain, which in turn prepares and constrains one's pursuit toward certain educational and occupational fields (Wang and Degol, 2013, 2017 for reviews).

Theories of career choice and development have also given personal work values a crucial role in one's educational and career choices (Holland, 1997; Eccles, 2009). Work or career values are the desired characteristics of one's current or future job and explain individual differences in vocational interests and career choices (Super, 1962; Judge and Bretz, 1992; Berings et al., 2004). Career choice is assumed to be made after various career options and their associated characteristics (e.g., money, social connect, family-balance) have been considered. These options are evaluated and identified as whether or not they align with one's personal goals, values, and preferences (Eccles, 2009). Although a large body of research using Eccles's expectancy-value theory has identified various personal work values or academic task values that contribute to gender disparities within the STEM fields, relatively few studies have examined the joint contributions of both critical sets of values in explaining STEM career choices (Wang et al., 2015; Eccles and Wang, 2016; see Wang and Degol, 2017, for a review). Furthermore, Eccles' and other value theories suggest that the *relative importance* of values matters most for guiding career pathways because choices of college major and career are made from a variety of options and their associated characteristics (Eccles, 2009). However, we are not aware of any study that has taken a person-oriented approach to examine the different intraindividual pattern of personal work values and its association with STEM participation.

To fill these gaps, this study examines how Finnish 11th-graders' personal work values and academic task values affect their actual career choices (6 or 8 years postsecondary school). We first examine the intraindividual patterns associated with students' ratings of the *relative importance* of work values across five domains (society, family, monetary, career prospect, and people-orientation) using a person-oriented approach. Second, we investigate the incremental effects of gender differences in work value profiles on the gender gap across STEM sub-disciplines (non-STEM vs. math-intensive vs. life science fields) above and beyond the established effects of academic task values. Finally, because career choice processes may vary across required educational levels, we test the generalizability of the predictive patterns across two educational levels of STEM professions: the professional and the support role levels STEM fields. This study therefore provides a comprehensive test of the psychological mechanisms proposed by Eccles' expectancy-value theory that underlie individual and gender differences in educational and occupational choices.

### Work values, STEM career choice, and gender differences

Work values have been at the center of several prominent theories of vocational choice and development (e.g., Super, 1990; Holland, 1997; Eccles, 2009). Over the past several decades, an enormous body of research has demonstrated that work values are one of the most important influences leading people to different occupations (Su and Rounds, 2015). However, work values have been somewhat overlooked in the literature relating to STEM occupational fields until recently (see Diekman et al., 2015, 2017 for reviews). To date, research has drawn on a variety of instruments and classification of work values (see Johnson et al., 2007 for a review). In this study, we reviewed recent studies on work values and STEM career choices and identified several work value types that are assumed to be related to gender differences in preferences that may affect STEM career choices.

#### Social values and working with others

Social values refer to valuing work that allows one to directly help people and contribute to society, which is highly related to *work with people* (i.e., a job that allows one to interact and help co-workers and work in teams). These two work value components have been elaborated in different theoretical frameworks, such as communal goals (e.g., Diekman et al., 2011, 2015), social interests [e.g., (Su et al., 2009; Su and Rounds, 2015); based on Holland's (1997) seminal work], and people-orientation (e.g., Woodcock et al., 2013). STEM fields are likely to deter people who endorse these social work values because these fields are often considered incompatible with goals of directly benefitting others, collaboration, or altruism (Diekman et al., 2015, 2017). Regardless of the theoretical framework used, research has shown that women prefer jobs where they can help and work with other people, whereas men prefer working with objects. Such gender differences are associated with the gender disparities in STEM fields (Su et al., 2009; Diekman et al., 2010, 2011; Woodcock et al., 2013). More recently, the gender differences in preferences of men and women to social work values are found to be useful to explain gender imbalance within STEM fields (i.e., life science vs. math-intensive; Su and Rounds, 2015; Wang et al., 2015; Eccles and Wang, 2016). Indeed, math-intensive fields involve a heavy thing-orientation component, whereas other STEM sub-disciplines such as medicine, nutrition, biology, and psychology science (life science) are more focused on working with and helping people and other living beings (Su and Rounds, 2015). Men and women who placed high value on having jobs associated with people and altruistic concerns were more likely to choose a life science rather than math-intensive career (e.g., Su and Rounds, 2015; Eccles and Wang, 2016). Importantly, gender differences in valuing working with people and altruism (favoring women) significantly explained why women are over-represented in STEM fields that are more people-oriented and less thing-oriented (i.e., life sciences; e.g., Su and Rounds, 2015; Eccles and Wang, 2016).

#### Material value and status

STEM fields are often considered more likely to provide opportunities for agentic (rather than communal) goal fulfillment (e.g., power, status, financial rewards; e.g., Brown and Diekman, 2010; Diekman et al., 2011). For example, even 6th graders (age 12) were found to be likely to associate science with power (Jones et al., 2000). STEM fields, particularly math-intensive fields, also dominate the list of top-earning college majors (Valla and Ceci, 2014). Material value and status have been well-documented in different theoretical conceptualizations of work values (e.g., Sagie and Elizur, 1996; Ros et al., 1999; Lyons et al., 2010). Material value is related to valuing work primarily for the salary or other compensation, and status refers to valuing work for its prestige, power, and authority (e.g., Ros et al., 1999). Research has shown that men tend to place more value on jobs that yield high income, power, and prestige compared to women (Eccles et al., 1999; Abele and Spurk, 2011). For instance, even 6- to 11-year-old boys showed greater interest than girls in professions recognized for their lucrative remuneration (Hayes et al., 2018). Such gender differences have been found to impede women's STEM pursuits (particularly in math-intensive fields, Eccles et al., 1999; Diekman et al., 2010, 2015).

#### Work-family balance (family value)

Work-family balance is another deterrent to women in STEM fields. Valuing work-family balance is directly related to gender role identity, with a traditional feminine identity leading one to place more emphasis on family and less on work and the reverse for a traditional masculine identity (Eccles, 2009). Compared to men, women are more willing to make occupational sacrifices for the family and prefer work-centered lifestyle at lower rates (Diekman et al., 2015; Wang et al., 2015; Wang and Degol, 2017). This gender difference emerges in late adolescence and young adulthood since men and women begin to consider their future more closely (Weisgram et al., 2010). Importantly, more recent research has found that adolescents and young adults perceive STEM careers afford family values less than other values such as money, power, and altruism^1^; the perception that science affords family values predicts interest in pursuing science studies/careers (Diekman et al., 2015; Weisgram and Diekman, 2017). Taken together, research has revealed that endorsement of work-family balance directed women away from masculine/STEM occupations (e.g., Frome et al., 2008; Ferriman et al., 2009; Weisgram et al., 2010; Diekman et al., 2015), particularly professional-level (e.g., scientist, Williams and Ceci, 2012; Mason, 2014) and math-intensive occupations (e.g., Computer Science, Ceci and Williams, 2011; Beyer, 2014).

While the evidence reviewed above documented that each single work value is associated with individual's career aspirations and choices, the relative hierarchical importance of these values may play a more critical role in clarifying one's perceptions, interests, and career goals (Jin and Rounds, 2012). The relative hierarchy of personal work values has been well elaborated in Eccles' expectancy-value theory (Eccles, 2009). Specifically, behavioral choices are assumed to depend upon a series of value-based calculations that weigh the relative (not absolute) subjective value across the variety of perceived available options associated with different occupational characteristics. For example, if one places a higher value on working with people than working with objects, machines, and tools, one is likely to prefer occupations that allow them to interact with people (e.g., life science and humanities). Thus, people's relative work values channel their educational and occupational decision-making and attainment. The extant studies, however, mainly focus on between-person differences in different work values, which limits our understanding of how individuals weigh up pros and cons for each option that leads to career choices.

Based on the literature reviewed above, in this study we focused on five core work values (a) Social value, (b) Working with people, (c) Material value (d) Status, and (e) Life-work balance. The inclusion of the five work values will enable us to examine the different intraindividual patterns across various core personal work values within sample and then to assess how these pattern groups contribute to gender differences in occupational choices related to STEM fields.

It is important to note that previous research has shown that work values stabilize by late adolescence, when students' intentions to pursue (or not to pursue) STEM majors are crystallizing (e.g., Jin and Rounds, 2012; Lechner et al., 2017). For example, Jin and Rounds (2012) conducted a meta-analysis study and showed that different work values (e.g., social values and status) are relatively stable from colleague years (ages 18–21.9) to young adulthood (ages 22–25.9). Similarly, Lechner et al. (2017) found such high rank-order and mean-level stability of work values of those aged between 20 and 25. These results are in line with a dynamic system perspective on work values development, which posits that individuals' value structure tends to become more stable and coherent with age (Vecchione et al., 2012). Although work values with STEM career were assessed at the same time point in this study, previous research (e.g., Bardi et al., 2014; Diekman et al., 2017) has suggested that people are more likely to choose their career transitions based on their values (self-selection processes) rather being socialized into their self-chosen careers (socialization). For example, Bardi et al. (2014) showed that in the transition to vocational training (of new police recruits) and to different university majors (psychology vs. business students), there were no significant value changes which would imply socialization effects. Taken together, the presence of self-selection processes and work values' high stability during postsecondary school transition support our hypothesis that work values guide individuals' choices toward (or away from) STEM careers from early life stages (see below).

### Incremental role of work values on career choices in high school

High school is a critical stage of adolescence when career aspirations began to crystallize on the basis of youth academic and career/work values (Eccles, 2009; Su et al., 2009). Youth are granted options to enroll in courses that are of interest, usefulness, and importance to them starting in high school, further creating a divide in STEM knowledge and learning experience between those who value and enroll in more advanced STEM courses, and those who de-value and opt out of challenging STEM courses. From a developmental perspective, Eccles (2009) hypothesized that individuals develop higher academic value for tasks and careers that they perceive as being closely aligned with their work values, leading them to preferentially select courses that are positively linked to their personal needs and identities. For example, a girl whose interests in activities that allow her to interact with and help others may choose to focus on classes and activities that fulfill her personal goals through a preference for those related to humanities. As such, she may come to place more academic task values on humanities than on other subjects. Both types of task values help her accumulate knowledge and skills associated with humanities and prepares her for entry into humanities-related majors or careers (Eccles, 2009; Lee et al., 2015).

Gender differences in task values attached to various academic subjects prevalent in STEM (e.g., math/science) and non-STEM fields (e.g., humanities, arts) are related to gendered educational and career aspirations and choices (Eccles, 2009; Wang and Degol, 2013, 2017). More specifically, men are likely to perceive math and physical science more important, useful, and enjoyable than women, whereas women are likely to have higher task values for language, social studies, and artistic subjects (e.g., arts, music; e.g., Chow et al., 2012; Wang et al., 2015; Eccles and Wang, 2016). These gendered differences in the academic task values partially contribute to overall underrepresentation of women in STEM fields (Chow et al., 2012; Guo et al., 2015) as well as the differential representation of gender across math-intensive and life science fields (Su and Rounds, 2015; Eccles and Wang, 2016).

While the relative hierarchy of work values and academic task values comprise essential parts of individuals' identity and can direct both men and women to different educational and career paths, their *joint* contributions to the prediction of career choices across STEM sub-disciplines (math-intensive and life science) have rarely been investigated. Given well-documented evidence as to the effect of academic values (Wang and Degol, 2013, 2017 for reviews), of particular interest in this study is to explore the incremental influence of work value profiles on career choices over and above academic values. Furthermore, recent research has stressed the need to distinguish between occupational choice processes for two fundamentally different occupational levels (professional- vs. support-level; Su and Rounds, 2015). For example, gender differences in interests in support-level life science careers (e.g., medical services) favoring women are larger than those in profession-level careers (e.g., medical science; Su and Rounds, 2015). In this study, to gain a better understanding about work values and academic task values that contribute to the differential participation of women across STEM sub-disciplines, career choices were operationalized into three categories: math-intensive, life science, and non-STEM occupations, and two social status groups: professional- and support- level occupations (see below for more details).

## The current study

The present investigation aims to examine the incremental contribution of the work value hierarchy in predicting individual and gender differences in STEM choices after accounting for academic task values. By taking into account both academic task values and work values as well as different domains and levels of occupations within STEM fields, this study provides a greater understanding of the motivational dynamics leading men and women to different STEM career pathways during transition into early adulthood. To achieve this aim, two overarching research questions are examined. For each research question, specific hypotheses (predictions) and empirical analysis questions are presented as follow.

**Overarching Research questions 1 (Q1): How does the relative work value hierarchy influence individual differences in STEM choices above academic task value?**

**Question 1a (Q1a)**. How many distinct hierarchical patterns (profiles) of work values will be captured? It is difficult to predict the exact number of groups that present the qualitatively and quantitatively distinct patterns of work values given the limited existing empirical research. As such, we leave it as an exploratory research question to be explored.
**Hypothesis 1 (H1a)**. We expect that work value profiles will significantly discriminate between people entering non-STEM, life science, and math-intensive fields above and beyond academic task values (Eccles, 2009; Su et al., 2009). While we are not able to propose clear a priori expectations as to the intraindividual patterns of work profiles, we are tempted to derive our hypotheses from previous findings related to each work value. As such, we expect that the groups where family value and social value/working with others are prioritized will be more likely to enter non-STEM fields rather than life science and particularly careers in math-intensive STEM fields (e.g., Ferriman et al., 2009; Diekman et al., 2011; Woodcock et al., 2013; Wang et al., 2015); the groups where money and career prospect (status) values are dominant will be more likely to pursue life science and particularly math-intensive careers over non-STEM careers, compared to other profile groups (e.g., Eccles et al., 1999; Abele and Spurk, 2011; Diekman et al., 2015).
**Question 1b (Q1b)**. Given the limited literature in comparisons between professional-level vs. support-level occupations within STEM fields, we leave as an open research question whether different predictive patterns merge for individuals entering professional-level vs. support-level STEM fields.

**Overarching Research Questions 2 (Q2): How does the relative work value hierarchy differ by gender and influence gender imbalance in STEM choices above academic task value?**

**Hypothesis 2a (H2a):** We anticipate that men will be over-represented in the profiles where monetary and career prospect are more highly endorsed than other values (i.e., social value, working with people, and family value), with the reverse being true for women (e.g., Diekman et al., 2015; Su and Rounds, 2015).
**Hypothesis 2b (H2b):** These gender differences will help explain gender imbalances in STEM choice above and beyond the gender differences in academic task values (Eccles, 2009; Wang and Degol, 2013, 2017).

## Methods

### Participants

**Figure 1:**
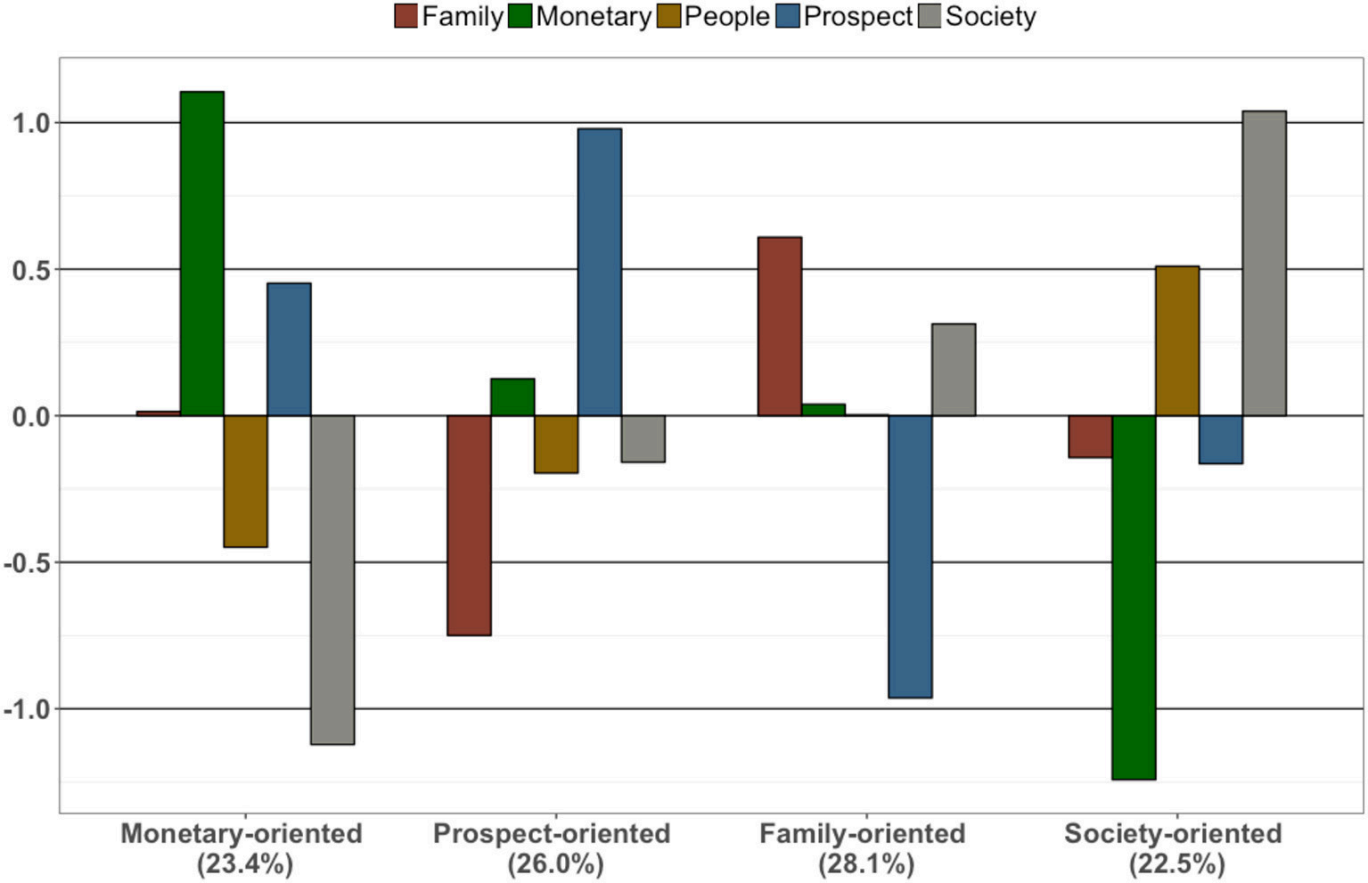
Work values profiles. Figure presented here is based on factor scores that were standardized within each individual. Percentages represent the proportion of population classified into the respective profiles.

The data set used in the present study is part of the larger Finnish Educational (FinEdu) Transition Studies. FinEdu is a multiple wave longitudinal follow-up study initiated in 2004 tracking two cohorts comprising 675 9th graders from nine comprehensive schools (Cohort 1, mean age = 16 years) and 584 11th graders from 13 upper secondary schools (Cohort 2, mean age = 18 years) in a medium-sized city in Middle Finland. Students were tracked every two years, through high school, higher education and employment. For this study, we utilized questionnaire data from both cohorts on academic task values when students were in 11th grade (2006 data for cohort 1 and 2004 data for cohort 2, total *N* = 1,259) as well as on their work values and STEM participation in the 2013 follow-up (cohort 1: 6 years after postsecondary school, mean age = 25 years; cohort 2: 8 years after postsecondary school, mean age = 27 years; total *N* = 892, 71% response rate, see Figure 1). Girls comprised 59.2% of the sample and almost all participants (99%) reported Finnish as their mother tongue.

### Finnish context

According to the last Programme for International Student Assessment (PISA) survey (OECD, 2016), Finnish 15-year-old adolescents had relatively high science and math performance (3rd and 8th across OECD countries, respectively). However, only 17% of them expressed their career aspirations in STEM fields (11% in life science fields), which was much lower than that in the U.S. (38%) and average OECD countries (25%). While Finland is a gender equality pioneer in terms of the low gender gaps in education, health, and economic/political participation and offers great gender equality in work/family policies, women are still overall under-represented in STEM fields (Esping-Andersen, 2002; Hausmann and Tyson, 2015). The Finnish egalitarian context, allowing equal possibilities for men and women to pursue STEM careers, provides a unique opportunity to investigate the underlying motivational mechanism that directs individual and gendered career development and choices.

### Measures

#### Academic task values

Academic task values in five school subject domains, including Finnish, math/science, humanities, foreign language, and practical subjects/arts, were measured by the task values scale developed from expectancy-value theory at Grade 11 (Eccles, 2009). The scale comprised three items “How interesting (important, useful) do you think each of the following subjects is?” to assess the interest, importance, and usefulness of each subject domain. All task values items were coded on a 7-point scale (from “not at all” to 7 “very much”). The domain-specific latent task values constructs demonstrated satisfactory reliability across time (0.78–0.86, see Appendix A in Supplementary Material for more details).

#### Work values

We used a set of 16 items derived from the Meaning of Work Study (MOW International Research Team, 1987) and Fit-Choice scale (Watt and Richardson, 2017) to measure five aspects of work values 6 or 8 years after postsecondary school (age 25 or 27 depending on cohorts). The items measured monetary (e.g., “the job allows me to earn a good salary), career prospect (e.g., “the job provides good opportunities for upgrading and promotion”), society (e.g., “the job allows me an opportunity to serve society”), family (e.g., “the job has hours that fit with family responsibilities”), and people-oriented work values (e.g., “the job allows me to work together with others”). Respondents rated the importance they attached to different job characteristics on a 7-point scale (from strongly disagree to strongly agree). Scale reliabilities for all work values were acceptable (0.81–0.90, see Appendix A in Supplementary Material for factor structure of the five work values).

#### STEM participation

Participants' STEM participation was measured 6–8 years after high school transition (ages of 25–27). It should be noted that a special feature of Finnish educational system is the high graduation age for academic track students in university. On average, the age of completion of university degree in Finland is between 25 and 28 years (Sortheix et al., 2015). As such, we assessed participants' actual STEM participation (i.e., studying or working in STEM fields) based on two questions: (1) “What is your field of study at the moment?” and (2) “What is your professional field at the moment?”. At that time point, 54% of participants had entered the workforce, for which we used question 2 to measure their actual STEM participation, otherwise, we used question 1. Supplemental multiple-group analysis indicates that separating those who have actually entered the workforce and those who are still in studying into two groups results in similar findings in relation to the prediction of STEM participation (see Appendix B in Supplementary Material). In this study, therefore, we focus on the results based on combining two groups to avoid complication.

We operationalized STEM occupations into two subsets: math-intensive and life science (see Eccles and Wang, 2016). Within STEM occupations, the categories below were further classified into support-level and professional-level occupations based on the skills and training required (Su and Rounds, 2015), which allows us to test the generalizability of the predictive patterns across two levels of STEM professions. Mathematics, Physical Sciences, Engineering, Computer Science, Health Sciences, Biological Science, Medical Science, and math and science teachers^2^ were categorized as the profession-level job, whereas Science Technicians, Engineering Technicians, Mechanics and Electronics and Medical services were categorized as the technical-level jobs (see Su and Rounds, 2015; OECD, 2016, p. 283 for the detailed classification, also see Appendix C in Supplementary Material for a classified list of majors/professions for the present study).

#### Demographic factors and matriculation scores

Gender was coded as 0 (women) or 1 (man). Parent occupational status was indicated by parents' occupations reported at Grade 11 (age 18). Matriculation examination results in Finnish and math were also included in this study. Given that students participated in university entrance exam at different time points, Matriculation examination scores, the only standardized testing in Finland throughout the whole educational career, were collected during the postsecondary school transition. Each participant only had one matriculation score for each subject (see Appendix D in Supplementary Material for more details).

### Analytic strategies

#### Missing data analysis

The missing completely at random (MCAR) test (Jamshidian et al., 2014) revealed that data was not missing complete at random, *p* < 0.01. To determine whether the students who participated in 11th grade differed from those who dropped out between the ages of 18 and 27, a series of independent samples contingency table analyses and *t*-tests were conducted with both demographic variables and other variables used in the analyses. We found men were significantly more likely to drop out of the study than women during the post-high school transition (*t* = 5.32, *p* < 0.00). Participants with lower GPA at Grade 11 (0.40 *SD*) were also significantly more likely to drop out of the study during the transition (*t* = 2.34, *p* < 0.00). It should be noted that missing data were not associated with work values and academic task values. In all analyses, we operated under the assumption that data were not MCAR but were missing at random. Full Information Maximum Likelihood estimation was used to cope with the missing data. Gender and GPA at Grade 11 were included as auxiliary variables in the data analyses [confirmatory factor analysis (CFA) and latent profile analysis (LPA)].

In the present study, analyses were conducted with Mplus 7.13 using the robust maximum likelihood estimator. First, we conducted a CFA to examine the factor structures of work values and academic task values. Subsequently, LPA, a person-oriented modeling technique, was used to identify characteristically distinct sub-populations of work values across the five domains. It assumes that there is an underlying categorical latent variable that characterizes an individual's class or profile based on the observed data (Muthén, 2001). LPA is a probabilistic model-based method in which estimated posterior probabilities of class membership are used to group individuals into latent classes. It should be noted that factor scores of the work values saved from preliminary measurement models were used in the LPA (Morin and Marsh, 2015). Particularly, we standardized five factor scores of work values within each individual before conducting LPA, which allowed us to disentangle shape differences from level effects (Morin and Marsh, 2015). Several indicators were used to select the optimal number of profiles (groups): the Akaike Information Criterion (AIC), the Consistent AIC (CAIC), the Bayesian information criterion (BIC), and the sample-adjusted BIC (SABIC). A lower value on these indicators suggests a better-fitting model. These information criteria should be graphically presented through “elbow plots” illustrating the gains associated with additional profiles (Morin and Marsh, 2015). In these plots, the point after which the slope flattens indicates the optimal number of profiles in the data. To further secure our decision in selecting the best model, we used the adjusted likelihood ratio test (LMR-LRT) and the bootstrap likelihood ratio test (BLRT) (Lo et al., 2001). Nonsignificant LMR-LRT and BLRT tests indicate that a model with *k-1* profile model would provide a better fit compared to a *k* profile model. Finally, we also relied on the Entropy Index that summarizes classification accuracy (Lubke and Muthén, 2007). The entropy varies from 0 to 1, with higher values indicating fewer classification errors. While there appears to be no definitive criteria for determining optimal numbers of latent classes when estimating LPA models, researchers have recommended the use of multiple statistical indices, along with conceptual considerations and interpretability of the latent groups (Morin and Marsh, 2015).

Second, based on the LPA results, a series of hierarchical regressions were conducted to explore how the work value profile memberships and academic task values predict STEM participation and gendered effect on STEM participation. Mixture models in Mplus provide class membership probabilities for each individual. Rather than using an “all-or-none” approach of assigning class membership to participants based on the highest probability for one of the profiles, we employed each individual's estimated probability of membership for each class as sampling probabilities (i.e., CPROB1-CPROB4 in SAVEDATA of Mplus output) to 25 created imputations of class membership and combined them with the original sample (Sahdra et al., 2017). The subsequent hierarchical regression analyses are based on 25 imputations in Mplus. All data analyses were run separately, and the results were aggregated appropriately in order to obtain unbiased estimates (Rubin, 1987). Thus, this approach allows us to account for classification uncertainty in the latent class membership^3^ and test mediation effects for gender.

The third step of our analyses focused on the question whether work value profiles and academic task values can explain the expected gender gap in STEM aspirations (Q3). We approached this question by testing how much the expected gender differences in STEM participation would be reduced by adding work value profiles and academic task values to the model using hierarchical regression analyses. That is, we calculated the magnitude of the relative indirect effects of gender (Huang et al., 2004; also see Wang et al., 2015), which can be loosely interpreted as the percentage reduction in the unstandardized regression coefficients (b) of gender between Model 1 (b_M1_) and Model 2 (b_M2_):

$$\Delta \text{b}=\frac{{\text{b}}_{\text{M1}}{-\text{b}}_{\text{M2}}}{{\text{b}}_{\text{M1}}}\times 100$$

## Results

### Preliminary CFA

Results showed that the CFA model, in which five work values and five subject-specific values were included, fit the data well [e.g., the comparative fit index (CFI) = 0.941, the Tucker–Lewis index (TLI) = 0.928, the root mean square error of approximation (RMSEA) = 0.042, see Appendix A in Supplementary Material for the factor structure).

### General descriptive results for gender

Two sets of mean testing of work and academic task values within- and between-gender differences were examined (see Appendix E in Supplementary Material). For within-gender comparison, women placed relatively high values on the Finnish subject and the lowest values on math and science; the reverse was true for men. Men placed relatively higher value on monetary rewards and career growth, whereas women placed relatively higher value on work-life balance than on other job characteristics. More women chose non-STEM than STEM occupations, whereas men were slightly overrepresented in STEM occupations. More women entered life science than math-intensive fields; men did the reverse. However, when only professional-level jobs were considered, the difference in entering life science vs. math-intensive fields became substantively smaller for women, but not for men.

For between-gender comparison, men had higher math and science values than women, whereas women had higher values than men in the other four academic domains. Men placed more value on salary and career prospect than women; women placed more value than men on family, working with people, and altruism. Within STEM, men were over-represented in math-intensive fields; women were over-represented, but to a lesser extent, in life science fields. The pattern of results was similar when only professional-level jobs were considered (see Appendix E in Supplementary Material).

### Classes description

The values for AIC, BIC, CAIC, and SABIC for the one- to seven-profile solutions continued to decrease with the addition of profiles (see Table 1). LMR-LRT became non-significant after four-profile solutions, whereas significant BLRT was showed from two- to seven-profile solutions. In accordance with previous recommendations, we also relied on elbow plots to help in the selection of the final solution. The elbow plot showed a relatively clear plateau at four profiles, after which improvement in fit became marginal (see Appendix F in Supplementary Material). Examination of the 4-profile solution shows them to be fully proper and interpretable. Examination of adjacent 3- and 5-profile solutions confirmed the added value of the 4-profile solution compared to the 3-profile solutions, and the lack of added value of the 5-profile solution which resulted in the estimation of additional very small profiles (4.1%) that brought no new information to the model (i.e., had the same shape as other profiles). Finally, entropy values suggested classification qualities for the models with four-profile solution was reasonable (0.858). More importantly, four distinct profiles provided substantive interpretation, in which monetary, prospect, family, and society values discriminate between profiles (e.g., Eccles, 2009; Diekman et al., 2015, 2017; Wang and Degol, 2017). Thus, we retained it as our final solution.

**Table 1:** Fit indices from LPA models.

**Model**	**#fp**	**LL**	**AIC**	**CAIC**	**BIC**	**ABIC**	**pLMR**	**pBLRT**	**Entropy**
1-Class	10	−8147.816	16315.631	16367.012	16335.247	16377.013	NA	NA	NA
2-Class	21	−7396.78	14835.559	14943.459	14876.753	14964.460	< 0.001	< 0.001	0.894
3-Class	32	−7061.118	14186.236	14350.655	14249.008	14382.654	0.004	< 0.001	0.884
4-Class	43	−6727.368	13540.736	13761.673	13625.085	13804.673	0.006	< 0.001	0.858
5-Class	54	−6655.016	13418.03	13749.49	13695.49	13523.96	0.076	< 0.001	0.858
6-Class	65	−6598.419	13326.84	13725.81	13660.81	13454.34	0.834	< 0.001	0.869
7-Class	76	−6548.516	13249.03	13715.53	13639.53	13398.11	0.832	< 0.001	0.881

*LL, Model log-likelihood; #fp, number of free parameters; AIC, Akaike's Information Criterion; CAIC, consistent AIC; BIC, Bayesian Information Criterion; ABIC, sample-size adjusted BIC; LMR, Lo-Mendell-Rubin likelihood ratio; BLRT, p-value for bootstrap likelihood ratio test*.

It should be noted that the values (means of each domain in each profile) presented in Figure 1 were based on factor scores that were standardized within each individual across the five work values. Thus, the histogram bars cannot be directly compared across different work value profiles. Profile 1 was characterized by relatively higher monetary importance, followed by traditional career prospects. This group also attached relatively less value on social contribution and working with people. We labeled this profile *Monetary-oriented*. It described 23.4% of the participants (see Table 2). In Profile 2 (26.1%), career prospect was rated the most important and family needs were rated as least important. Thus, we labeled this group *Prospect-oriented*. In contrast, Profile 3 (28.1%) rated family values the most important and career prospects the least important; we labeled this group *Family-oriented*. Profile 4 (22.5%) rated society contribution and working with people the most important and monetary rewards the least important; we labeled it *Society-oriented*.

**Table 2:** Gender distribution across the four work value profiles.

	**P1 *Monetary-oriented N* = 295 (23.4%)**	**P2 *Prospect oriented N* = 329 (26.1%)**	**P3 *Family-oriented N* = 355 (28.1%)**	**P4 *Society-oriented N* = 280 (22.5%)**	**Mean test between profiles**
	**Mean (SE)**	**Mean (SE)**	**Mean (SE)**	**Mean (SE)**	***F***_(3, 1259)_
Gender distribution					55.36^***^
Women	123 (16.5%)^a^	200 (26.8%)^a^	224 (30.1%)^a^	198 (26.6%)^a^	
Men	172 (33.5%)^b^	129 (25.1%)^b^	131 (25.5%)^b^	82 (16.0%)^b^	

*** *p < 0.001*.

a The percentage of women;

b *The percentage of men*.

### Prediction of stem participation

#### Prediction of math-intensive and life science participation

We conducted hierarchical logistic regression to determine which variables discriminate between people who entered life science, math-intensive, and non-STEM careers. In the first set of regression models, we only included gender, parent occupational status, and matriculation scores as the predictors (see Table 3). Gender significantly predicted STEM occupation across three-pair comparisons. Math matriculation scores had a small effect on entry into math-intensive over life science and non-STEM fields.

**Table 3:** Hierarchical logistic regression predicting individuals' participation in math-intensive and life science fields.

**Predictors**	**STEP 1**						**STEP 2**					
	**Non-STEM vs. Math-intensive**		**Life science vs. Math-intensive**		**Life science vs. Non-STEM**		**Non-STEM vs. Math-intensive**		**Life science vs. Math-intensive**		**Life science vs. Non-STEM**	
	**coef**	**OR**	**coef**	**OR**	**coef**	**OR**	**coef**	**OR**	**coef**	**OR**	**coef**	**OR**
Gender	−2.03 (0.22)^***^	0.13	−2.50 (0.27)^***^	0.08	−0.47 (0.20)^*^	0.63	−1.75 (0.23)^***^	0.17	−2.44 (0.27)^***^	0.09	−0.69 (0.21)^**^	0.50
Parent occupational status	0.24 (0.13)	1.27	0.30 (0.16)	1.48	0.15 (0.11)	1.16	0.21 (0.13)	1.23	0.30 (0.16)	1.42	0.14 (0.11)	1.15
Finnish matriculation	0.01 (21)	1.01	0.11 (0.26)	1.12	0.11 (0.18)	1.12	0.01 (0.21)	1.01	0.12 (0.26)	1.13	0.11 (0.18)	1.12
Math matriculation	−0.36 (0.11)^**^	0.70	−0.44 (0.13)^**^	0.64	−0.19 (0.20)	0.83	−0.22 (0.20)	0.80	−0.32 (0.27)	0.73	−0.20 (0.21)	0.82
**ACADEMIC TASK VALUES**												
Finnish							−0.08 (0.15)	0.73	0.13 (0.17)	1.19	0.21 (0.12)	1.35
Math and Science							−0.75 (0.13)^***^	0.47	−0.23 (0.14)	0.79	0.52 (0.10)^***^	1.68
Humanities							0.69 (0.15)^***^	1.99	0.18 (0.15)	1.20	−0.51 (0.12)^***^	0.60
Foreign language							0.22 (0.13)	1.24	−0.01 (0.13)	0.99	−0.23 (0.11)^*^	0.79
Practical subjects and arts							0.03 (0.12)	1.03	0.10 (0.13)	1.11	0.07 (0.10)	1.07

* *p < 0.05*,

** *p < 0.01*,

*** *p < 0.001*.

Second, we added five subject-specific task values, which significantly predicted individual differences in the choice of non-STEM vs. life science and math-intensive fields (see Table 3). Consistent with previous research, math/science values were positively associated with entry into life science over non-STEM fields, whereas humanities values were positively associated with entry into non-STEM over life science fields. Similarly, math/science values were positively associated with entry into math-intensive over non-STEM fields. Academic values in humanities and foreign language were positively associated with entry into non-STEM over math-intensive fields. However, individual differences in the academic values did not explain differences in entry into life science vs. math-intensive fields.

Finally, we added the four work value profiles into the hierarchical regression model predicting STEM choices, controlling for all the variables previously included in the model (see Table 4). We also report odds ratios, reflecting the change in likelihood of entering life science vs. math-intensive fields associated with people being in a target work value profile vs. a comparison profile. For example, an OR (Odds Ratio) of 3 suggests that individuals in a target profile (compared to a comparison profile) are three times more likely to enter life science over math-intensive fields. As expected (H1a), individuals from the Society-oriented group had the greatest likelihood of choosing life science over math-intensive fields, followed by those from the Family-oriented group. In line with this, individuals in the Society-oriented group were more likely to enter non-STEM over math-intensive fields, compared to those from other groups. Individuals in the Prospect-oriented and Monetary-oriented groups were more likely to enter math-intensive fields over non-STEM fields, compared to individuals in other profiles. Both Prospect- and Monetary-oriented groups had a small and similar likelihood of pursing life science over math-intensive fields, however, the work value profiles were not significant predictors of entry into life science vs. non-STEM fields.

**Table 4:** Work value profiles and academic task values predicting individuals' participation in math-intensive and life science fields (Con't).

**Predictors**	**STEP 3**					
	**Non-STEM vs. Math-intensive**		**Life science vs. Math-intensive**		**Life science vs. Non-STEM**	
	**coef**	**OR**	**coef**	**OR**	**coef**	**OR**
Gender	−1.21 (0.34)^***^	0.30	−1.84 (0.28)^***^	0.16	−0.63 (0.21)^**^	0.53
Parent occupational status	0.21 (0.14)	1.23	0.29 (0.16)	1.45	0.16 (0.12)	1.17
Finnish matriculation	0.02 (0.20)	1.02	0.09 (0.24)	1.09	0.11 (0.19)	1.12
Math matriculation	−0.10 (0.20)	0.90	−0.30 (0.19)	0.69	−0.26 (0.21)	0.77
**ACADEMIC TASK VALUES**						
Finnish	−0.08 (0.16)	0.99	0.08 (0.17)	1.08	0.16 (0.12)	1.28
Math and Science	−0.70 (0.14)^***^	0.50	−0.16 (0.15)	0.85	0.54 (0.13)^***^	1.55
Humanities	0.73 (0.17)^***^	2.08	0.24 (0.16)	1.27	−0.49 (0.14)^***^	0.68
Foreign language	0.26 (0.14)	1.27	0.02 (0.14)	1.02	−0.24 (0.12)^*^	0.80
Practical subjects and arts	−0.03 (0.13)	0.97	0.03 (0.14)	1.03	0.06 (0.11)	1.06
**PROFILES**						
Vs. P1 (Monetary-oriented)						
P2 (Prospect-oriented)	0.24 (0.28)	1.27	0.21 (0.33)	1.51	−0.03 (0.27)	1.19
P3 (Family-oriented)	0.66 (0.28)^*^	1.93	1.09 (0.34)^**^	2.97	0.43 (0.26)	1.54
P4 (Society-oriented)	1.41 (0.42)^**^	4.10	1.91 (0.47)^***^	6.75	0.50 (0.28)	1.65
Vs. P2 (Prospect-oriented)						
P3 (Family-oriented)	0.42 (0.29)	1.52	0.68 (0.32)^*^	1.97	0.26 (0.23)	1.30
P4 (Society-oriented)	1.17 (0.41)^**^	3.22	1.50 (0.44)^**^	4.48	0.32 (0.24)	1.38
Vs. P4 (Society-oriented)						
P3 (Family-oriented)	−0.75 (0.36)^*^	0.47	−0.82 (0.37)^*^	0.45	−0.07 (0.22)	0.93

* *p < 0.05*,

** *p < 0.01*,

*** *p < 0.001*.

Results also showed that the effects of academic task values remain significant after including work values profiles in the model. A more detailed examination of the relations between academic task values and work value profiles are presented in Appendix G in Supplementary Material.

#### Profession-level vs. support-level STEM fields

We ran the regressions separately for individuals entering professional- and support-level STEM fields to compare the predictive pattern for entering professional-level STEM vs. non-STEM fields, with that for entering support-level STEM vs. non-STEM fields (see Table 5). Three major differences emerged. Firstly, the *Society-oriented* group had a greater likelihood of attaining support-level life science rather than support-level math-intensive careers, followed by the *Family-oriented* group. Second, the *Society- and Family-oriented* groups were more likely to pursue support-level life science over non-STEM careers compared those in the other profiles. Finally, compared to the *Family-oriented* group, the *Society-oriented* group was more likely to enter non-STEM vs. support-level math-intensive fields, whereas such an effect was insignificant when looking at entering non-STEM vs. profession-level math-intensive fields.

**Table 5:** Separate groups regression predicting STEM participation for individuals entering profession-level vs. support-level fields.

**Predictors**	**Profession-level**						**Support-level**					
	**Non-STEM vs. Math-intensive**		**Life science vs. Math-intensive**		**Life science vs. Non-STEM**		**Non-STEM vs. Math-intensive**		**Life science vs. Math-intensive**		**Life science vs. Non-STEM**	
	**coef**	**OR**	**coef**	**OR**	**coef**	**OR**	**coef**	**OR**	**coef**	**OR**	**coef**	**OR**
Gender	−1.11 (0.30)^**^	0.33	−1.95 (0.48)^**^	0.14	−0.61 (0.23)^*^	0.54	−1.53 (0.42)^***^	0.22	−1.65 (0.45)^***^	0.19	−0.51 (0.24)^*^	0.60
Parent occupational status	0.12 (0.19)	1.13	0.24 (0.18)	1.27	0.42 (0.24)	1.52	0.23 (0.18)	1.26	0.32 (0.24)	1.38	0.14 (0.14)	1.15
Finnish matriculation	−0.12 (0.23)	0.89	0.35 (0.36)	1.42	0.47 (0.31)	1.60	0.12 (0.31)	1.13	0.15 (0.35)	1.16	0.03 (0.22)	1.03
Math matriculation	−0.23 (0.24)	0.79	−0.12 (0.39)	0.89	0.11 (0.37)	1.12	0.14 (0.33)	1.15	−0.35 (0.39)	0.70	−0.49 (0.26)	0.61
**ACADEMIC TASK VALUES**												
Finnish	−0.53 (0.18)^**^	0.59	−0.21 (0.27)	0.81	0.31 (0.13)^*^	1.36	0.07 (0.21)	1.07	0.22 (0.23)	1.38	0.15 (0.11)	1.28
Math and Science	−1.00 (0.17)^***^	0.37	0.09 (0.26)	1.09	1.09 (0.22)^***^	2.97	−0.45 (0.17)^**^	0.64	−0.03 (0.19)	0.97	0.42 (0.11)^***^	1.52
Humanities	0.71 (0.17)^***^	2.03	0.14 (0.26)	1.15	−0.56 (0.24)^*^	0.57	0.78 (0.20)^***^	2.18	0.34 (0.20)	1.40	−0.44 (0.12)^***^	0.64
Foreign language	0.14 (0.17)	1.15	−0.27 (0.19)	0.76	−0.41 (0.14)^**^	0.66	0.35 (0.14)^*^	1.42	0.12 (0.16)	1.13	−0.23 (0.11)^*^	0.79
Practical subjects and arts	0.08 (0.14)	1.08	−0.01 (0.19)	0.99	−0.10 (0.16)	0.90	−0.14 (0.17)	0.87	−0.06 (0.19)	0.94	0.08 (0.10)	1.08
**PROFILES**												
Vs. P1 (*Monetary-oriented*)												
P2 (*Prospect-oriented*)	0.45 (0.34)	1.57	0.22 (0.49)	1.25	0.23 (0.42)	1.26	0.25 (0.39)	1.28	0.61 (0.47)	1.84	0.36 (0.33)	1.43
P3 (*Family-oriented*)	0.94 (0.36)^**^	2.56	0.22 (0.55)	1.25	0.73 (0.49)	2.08	0.50 (0.37)	1.65	1.31 (0.45)^**^	3.71	0.81 (0.32)^*^	2.25
P4 (*Society-oriented*)	1.27 (0.51)^*^	3.56	0.92 (0.69)	2.51	0.35 (0.51)	1.42	1.58 (0.62)^*^	4.85	2.51 (0.66)^**^	8.30	0.93 (0.34)^*^	2.53
Vs. P2 (*Prospect-oriented*)												
P3 (*Family-oriented*)	0.50 (0.38)	1.65	0.00 (0.53)	1.00	0.50 (0.44)	1.65	0.25 (0.40)	1.28	0.70 (0.33)^*^	2.01	0.45 (0.26)	1.57
P4 (*Society-oriented*)	0.82 (0.40)^*^	2.27	0.70 (0.66)	2.01	0.12 (0.48)	1.13	1.32 (0.52)^*^	3.42	1.90 (0.54)^**^	6.68	0.57 (0.30)	1.60
Vs. P4 (*Society-oriented*)												
P3 (*Family-oriented*)	0.33 (0.51)	1.39	−0.70 (0.70)	0.50	0.38 (0.52)	1.46	1.07 (0.52)^*^	2.91	−1.20 (0.53)^*^	0.30	0.12 (0.24)	1.03

* *p < 0.05*,

** *p < 0.01*,

*** *p < 0.001*.

#### Gender effects

As seen in Table 3 (also see Appendix E in Supplementary Material), there are significant gender differences in work value profiles and mean-level academic task values. Men were over-represented in the *Monetary-oriented* profile, whereas women were over-represented in the *Family- and Society-oriented* profiles. Men placed relatively high values on math and science and the lowest values on Humanities and languages. As predicted (H2a), academic task values explained (2.03–1.75)/2.03 = 14% of the gender differences (i.e., the relative indirect effects of gender) in entering non-STEM vs. math-intensive fields (comparing STEP1 to STEP2 in Table 4). When work value profiles and academic values were included, both sets of values explained (2.03–1.21)/2.03 = 40% (comparing STEP1 to STEP3, in Table 5) of gender differences in entering non-STEM vs. math-intensive fields. Taken together, results indicated that work value profiles further explained 40–14% = 26% of gender differences in entry into non-STEM over math-intensive fields, controlling for academic values. Similarly, results also indicated that further inclusion of work value profiles in the hierarchical regression model (STEP3) explained a notable proportion of gender differences in entering life science vs. math-intensive fields (24%), however, work value profiles or academic and task values did not close the gender gap in entering life science vs. non-STEM fields (the gender effects got slightly larger between STEP1 and STEP3).

## Discussion

Table 6 summaries key findings of the present study and indicates whether these findings supported our expectations. By employing a person-oriented approach and incorporating two crucial sets of predictors of STEM participation (work values and academic task values), the findings provided evidence of how intraindividual patterns of work values contribute to the gendered career pathways above and beyond academic task values.

**Table 6:** Summary for the key findings.

	**Hypothesis**	**Support for predictions**	**Inconsistent with predictions**	**Leave as research questions**
**Q1**	How does the relative work value hierarchy influence individual differences in STEM choices above academic task value?	**H1a:** work value profiles significantly discriminate between people entering STEM fields. Likelihood of choosing Non-STEM vs. math-intensive fields: society > family > prospect = monetary; Likelihood of choosing life science vs. math-intensive fields: society > family > prospect = monetary.	The work value profiles did not predict entry into life science vs. non-STEM fields.	**Q1a:** Number of work value profiles. Four profiles (monetary-, prospect-, family-, and society-oriented); **Q1b:** Predictions for comparison between professional-level vs. support-level STEM fields. Likelihood of choosing support-level life science vs. math-intensive fields: society > family > prospect = monetary; Likelihood of choosing support-level life science vs. non-STEM fields: society = family > monetary; Likelihood of choosing non-STEM vs. support-level math-intensive fields: society > family
**Q2**	How does the relative work value hierarchy differ by gender and influence gender imbalance in STEM choices above academic task value?	**H2a:** Gender distribution in profiles: Women are over-represented in family- and society-oriented groups; Men were over-represented in the Monetary-oriented profile **H2b:** Mediation effects Work value profiles were partially mediated gender differences (favoring men) in entering math-intensive vs. life science and non-STEM fields.	Work value profiles or academic and task values did not close the gender gap in entering life science vs. non-STEM fields.	

One of the unique contributions of the current study is identifying four relative-priority profiles of core personal work values and linking them to long-term STEM participation (6 or 8 years postsecondary school). As hypothesized, individuals in the *Family*- and *Society*-groups moved toward life science and non-STEM occupational pathways. Conversely, those in the *Prospect*- and *Monetary*-oriented groups moved toward math-intensive rather than other fields. These findings are inconsistent with previous studies based on a variable-oriented approach showing that work values placed on monetary rewards and career-focus did not predict STEM participation when controlling for other work values (Wang et al., 2015; Eccles and Wang, 2016). A potential explanation is that previous studies focused on between-person differences in work values and found that students tended to rate monetary/career-focused values similarly (indicated by smaller standard deviations, see Wang et al., 2015 for more details) relative to other work values, thus leading to insignificant effects on STEM aspirations. These between-person differences in work values could mask individual decision-making processes in career choices. Instead, using a person-oriented approach allows us to assess the nuanced details about how individuals prioritize different work values and weight different options. Students who place importance on potential earned income and career prospects than other work values would move toward math-intensive fields that dominate the list of top-earning college majors and yield more predictable career advancement pathways (Valla and Ceci, 2014).

However, inconsistent with our expectations, work value profiles did not differentiate between those who entered life science vs. non-STEM fields. By further exploring the predictive pattern for individuals entering professional- and support-level STEM fields, results indicate that individuals in the family- and society-oriented profiles are more likely to move toward support-level life science vs. non-STEM fields (but these profiles did not discriminate among those choosing profession-level life science vs. non-STEM fields). Indeed, support-level life science occupations may be perceived to more directly link to working with people as well as to be more time flexibility and fewer professional responsibilities, such as counselors and nurses, compared to profession-level life science (e.g., bioengineers, epidemiologist) and non-STEM fields (Kimmel et al., 2012). These occupational characteristics make support-level life science careers more attractive for individuals prioritizing social and family values. Following the same motivational mechanism, being in the *Family-oriented* or *Society-oriented* groups increased the likelihood of choosing a support-level life science rather than math-intensive careers (see Table 6). In contrast, work value profiles did not predict differences at the professional-level between life science vs. math-intensive choices, indicating that young adults are likely to believe that professional-level STEM careers have similar characteristics, such as entailing similar level of family-work balance conflict (e.g., Diekman et al., 2015; Eccles and Wang, 2016). These distinctions in the prediction of STEM participation provide a greater understanding of how work values motivate men and women to enter different STEM sub-disciplines and underscore the importance of assessing pathways leading to profession- and support-level STEM fields separately.

Consistent with previous studies (Wang and Degol, 2013, 2017), high task values in math and science coupled with low values in humanities moved both genders toward STEM career pathways, however, academic task values did not differentiate between those people going into life science vs. math-intensive. A possible explanation is that math and science values were measured as a single motivational construct, while different STEM occupations require different levels of math cognitive ability and domain-specific science skills. More recent studies have shown that high school students can distinguish academic values in math and different science domains, although students appear to have similar levels of achievement in those subjects (e.g., Guo et al., 2017).

The pattern of results regarding the gender gaps in task values and work values and their contribution to gendered career pathways herein were largely aligned with our expectations. Women' participation in STEM was partially influenced by the ways they prioritize different core personal work values and view different academic subjects, according to their personal goals and identities. Women viewed working with and helping people, and committing to family responsibilities, as important personal goals in the same way they placed high values on humanities in high school. Both value beliefs increased the likelihood of women moving away from math-intensive classes and activities in school. The opposite is true for men. For both genders, these processes constrain their options in educational and occupational pursuits of STEM and non-STEM fields, however, gender difference in both sets of value beliefs did not explain entry into life science vs. non-STEM fields. This suggests that women generally may believe that life science careers are congruent with their personal goals, values, and preferences, as are the values of non-STEM careers, which may help explain why women are over-represented in life science fields. Thus, more research is needed to include other social-psychological factors (e.g., gender-related stereotypes and biases) and provide a comprehensive picture of why such gender differences exist and how they are developed.

### Limitations and further research

Several limitations and caveats of this study must be noted. First, we did not include academic ability self-concept (i.e., expectancy of success), a variable that has been shown to significantly predict STEM participation (Wang and Degol, 2013), even though academic task values are better predictors of choice behaviors (Wang and Degol, 2013). Additionally, as mentioned earlier, math and science task values were operationalized as a single construct, which substantially limited our ability to detect motivational mechanism channeling people to different STEM sub-disciplines. Thus, the further inclusion of multiple domain-specific science expectancies and task values would provide a more comprehensive understanding of the roles of motivational beliefs in shaping career pathways.

Third, the current findings are correlational and no causal inferences should be made. Particularly, the data related to core personal work values and STEM participation were collected within a single wave. Although some of work value scales^4^ (i.e., monetary, career prospects) included in this study have been shown to be highly stable during postsecondary school transition (from age 20 to 25) based on the same sample (Lechner et al., 2017), the current data still does not allow us to examine the stability of the four work value profiles and their long-term prediction of STEM participation across time. Indeed, the transition from education to employment requires individuals to invest in new social roles and to adapt their behaviors and motivations to these new roles' requirement. Even though entering the workforce would potentially affect one's work values, recent studies have shown that family and work transitions have very small and limited effects on work values, especially when compared to stable background characteristics such as gender and family socioeconomic status (Sortheix et al., 2015; Lechner et al., 2017). Still, the robustness of our findings could be strengthened by carefully constructed longitudinal panel studies and experimental interventions to better understand the causal mechanisms in the career decision-making process.

Fourth, given that different fields within non-STEM fields involve different levels of earning prospects, social interaction, and math-intensity (e.g., economic sciences vs. archeology), it would be beneficial to further differentiate fields across non-STEM sub-disciplines and explore how work value profiles contribute to individual and gender differences in entry into different career fields.

Fifth, the participants of this study were drawn from central Finland, which did not allow to examine and compare the roles of socio-cultural and national differences in family, school, and work environment. For example, nations also differ in the perception of the gendered stereotypes linked to STEM and non-STEM occupations (Eccles and Wang, 2016). Thus, the cross-cultural variations in socialization and gender-role processes that influence choices of occupational pathways indicate that more comparative studies in more diverse settings are needed to advance our understanding of career choices.

Lastly, this study found that gender imbalance in the Society-oriented profile (favoring women) significantly contribute to gender differences in STEM representations. Relatedly, Baron-Cohen (2003) offered a similar explanation from a biological perspective, arguing that men and women have different brain types and women are better at empathizing (vs. systematizing) and more interested in careers involving social relations. However, the extent to which such gender differences reflect genetically based or hormonally based biological process, or social cultural processes, or more likely the interaction between these two broad types of developmental forces is not the focus of our paper and cannot be determined with the kind of data we have. Given the very classic nature of this debate, we encourage researchers to pursue this issue.

### Implications and practice

Despite these limitations, the current study has implications for intervention and practice. Firstly, the findings suggest that individuals in the *Monetary-oriented* groups (those who placed the least importance on social value) have the greatest likelihood of choosing math-intensive careers. This, however, does not mean that interventions aim at fostering math/science-course participation should focus on promoting student's monetary values, which are more highly valued by men. Rather, to reduce the gender gap in math/science-course taking, STEM educators could place greater emphasis on demonstrating the societal relevance of math-intensive skills and careers as well as how math-intensive fields can be collaborative and beneficial to society and have the ability to improve people's lives (Diekman et al., 2015; Wang and Degol, 2017). By doing this, we may be able to make STEM careers, particularly for math-intensive careers, more relatable and accessible to women in everyday life and thus attract more women to participate in these types of STEM activities (Valla and Ceci, 2014; Su and Rounds, 2015). At the same time, special efforts should also be made to ensure that students are well-informed of the whole variety of occupational options in STEM fields and what characteristics are attached to those occupations. This information enables women and men to better relate their personal goals and identities to different STEM careers.

Second, relatively high family-value coupled with relatively low career prospect work value is another key factor directing people away from math-intensive careers. Given the STEM labor market shortages, interventions promoting women's career ambition and helping them view potential bright future in STEM careers might be useful in recruiting science-talented and capable women to embark on STEM career paths, particularly in math-intensive fields. Additionally, integrating more family-friendly workplace policies in math-intensive fields might make these professions more enticing to people wanting better work-life balance. Such policy moves could counter the stereotype that math-intensive careers are more time demanding and higher work commitment than non-STEM careers (Diekman et al., 2015). This conception comes into direct conflict with women's work-life balance. Interventions designed to eliminate this conception might be an effective way to increase women's participation in math-intensive fields. Interestingly, such a perceived stereotype does not exist within life science and non-STEM fields.

Third, high math and science task values help to propel students toward STEM pathways. Interventions designed to increase students' perceptions of the relevance of math and science to their lives through teachers and parents have been found to be effective in triggering students' interest and academic performance in STEM topics (Lazowski and Hulleman, 2016). Other interventions focusing on positive school experience in relation to science, such as providing increased exposure to women scientist role models and challenging stereotypes of science masculinity, have been also proved to be useful in promoting women's motivation and engagement in STEM activities (Wang and Degol, 2017).

Finally, given that both work values and academic task values significantly contribute to gender differences in STEM fields, we call for more interventions that target both types of value beliefs and seek to enhance students' perception of value beliefs attached to activities and careers based on a long-term longitudinal design. Importantly, these interventions should be implemented at early ages since academic and work values are closely associated with ability development, academic engagement, and STEM educational and occupational preparedness (Eccles, 2009). On the other hand, we think it is important to think carefully about using our findings to socially engineer the next generations' career choices. Ideally, individuals should be helped to make the best career choices they can for themselves, influenced as little as possible by stereotypes, gendered patterns of socialization, and government policy. To say we would like women to be just as likely as men to consider becoming an engineer or a nurse or a medical doctor is one thing; to say we want to persuade a particular women or man to become a computer scientist rather than a journalist or professional musician is quite another.

## Ethics statement

The study was consistent with the ethical principles of human subjects. First, we told the detailed content of the study to the participants. Second, participants signed the informed consent on a voluntary basis.

## Author contributions

JG conceived of the study, performed the statistical analysis, and wrote up the paper; JE conceived of the study, provided suggestions for the statistical analysis, and commented the manuscript; FS participated in the design of the study and commented the manuscript; KS-A conceived of the study, participated in the coordination of the study, and commented the manuscript. All authors read and approved the final manuscript.

### Conflict of interest statement

The authors declare that the research was conducted in the absence of any commercial or financial relationships that could be construed as a potential conflict of interest.

## Footnotes

^1^The reason why STEM careers are perceived as being family “un-friendly” may be due to the overall stereotype of scientists, media depictions of scientists, and prior experience of science activities in school (Weisgram and Diekman, 2017, also see Diekman et al., 2015 for a review).

^2^In this study STEM occupations were defined based on the skills and training required. Given that in Finland math and science teachers require master's degrees specializing in math or a specific science domain, we categorized this occupation as part of the STEM careers, which is what has been defined in the U.S. (e.g., Miller et al., 2015; Wang et al., 2015).

^3^Note that a three-step approach implemented in Mplus also allows us to control for measurement error in profile membership (Asparouhov and Muthén, 2014). However, only DCATEGORICAL command is available for categorical distal outcomes (i.e., STEM participation) in the three-step approach (Muthén and Muthén, 1998-2012, p. 557). But DCATEGORICAL cannot be combined with other continuous covariates in LPA. In other words, it is unfeasible to examine the effect of class membership on STEM participation while controlling for the continuous covariates (e.g., demographic factors and academic task values) using the three-step approach with DCATEGORICAL (even using the manual three-step approach proposed in Asparouhov and Muthén, 2014). Thus, an alternative approach accounting for classification uncertainty was used in this study.

^4^Another three work value scales (i.e., Family-work balance, social values, working with others) included in this study was *only* assessed when participants are at age 25 (Cohort 1) or age 27 (Cohort 2), and thus we are not able to test the stability of these three constructs over time.
