# Supplementary material for: Gendered Pathways Toward STEM Careers: The Incremental Roles of Work Value Profiles Above Academic Task Values
Source: Front Psychol. 2018 Jul 2;9:1111. doi: 10.3389/fpsyg.2018.01111 (PMC6050506; doi:10.3389/fpsyg.2018.01111)
Supplement: Supplementary file 1 [file Table_1.DOCX]

*Online Supplemental Materials for:*

**Gendered Pathways Towards STEM Careers:**

**The Incremental Roles of Work Value Profiles Above Academic Task Values**

**Table of Contents**

[External Appendix A: Factor structure and Reliability of Five Work Values and Academic Task Values 2](#_Toc500533092)

[External Appendix B: Work Value Profiles and Academic Task Values Prediction (separating who entering workforce and studying university) 3](#_Toc500533093)

[External Appendix C: A Classified List of Majors/Professions for the Present Study 4](#_Toc500533094)

[External Appendix D: Demographic Factors and Matriculation Scores 5](#_Toc500533095)

[External Appendix E: Descriptive Statistics of the Sample and Tests of the Difference between Female and Male 6](#_Toc500533096)

[External Appendix F: Class Enumeration Process 7](#_Toc500533097)

[External Appendix G: Relations between Work Value Profiles and Subject-Specific Task Value 8](#_Toc500533098)

# External Appendix A: Factor structure and Reliability of Five Work Values and Academic Task Values

Table A1

*Factor structure and Reliability of Five Work Values and Academic Task Values*

| Item | Factor loadings | Reliability |
| --- | --- | --- |
| Monetary value |  |  |
| good pay | .82 | .89 |
| earns a good salary. | .97 |  |
| Prospect value |  |  |
| good opportunities for upgrading and promotion. | .85 | .90 |
| I can proceed on my career. | .90 |  |
| provides a clear pathway for career development. | .86 |  |
| Society value |  |  |
| allows me an opportunity to serve society | .79 | .87 |
| has possibilities for societal influence. | .80 |  |
| allows me to 'give back' to society. | .85 |  |
| allows me to help the underprivileged. | .73 |  |
| Family value |  |  |
| it has hours that fit with family responsibilities. | .52 | .81 |
| fits vacation time with family commitments. | .88 |  |
| work that is flexible (e.g. part-time options). | .81 |  |
| Work with others |  |  |
| work where I can work together with others. | .75 | .87 |
| work that allows me to help and support my co-workers. | .59 |  |
| involves working as part of a team. | .89 |  |
| offers a team-based work environment. | .89 |  |
| Task values in Finnish/math and science/ Humanistic/foreign language/ Arts and practical subjects |  | .83/.78/.85/.85/.86 |
| How interesting do you think each of the following subjects is | .88/.94/.91/.88/.89 |  |
| How important do you think each of the following subjects is | .82/.88/.89/.88/.86 |  |
| How useful do you think each of the following subjects is | .69/.67/.66/.59/.69 |  |

# External Appendix B: Work Value Profiles and Academic Task Values Prediction (separating who entering workforce and studying university)

Table B1

*Work Value Profiles and Academic Task Values Predicting Individuals’ Participation in Math-Intensive and Life Science Fields (separating who entering workforce and studying university)*

|  | Participants entering workforce | | |  | Participants studying university | | |
| --- | --- | --- | --- | --- | --- | --- | --- |
|  | Non-STEM vs.  Math-intensive | Life science vs.  Math-intensive | Life science vs.  Non-STEM |  | Non-STEM vs.  Math-intensive | Life science vs.  Math-intensive | Life science vs.  Non-STEM |
| Predictors | coef | coef | coef |  | coef | coef | coef |
| Gender | -1.35(.30)*** | -1.86(.33)** | -.52(.26)* |  | -1.15(.34)*** | -1.76(.37)*** | -.61(.30)* |
| Parent occupational status | .25(.18) | .31(.23) | .06(.18) |  | .15(.24) | .35(.27) | .20(.17) |
| Finnish matriculation | .04(.26) | -.05(.34) | -.09(.30) |  | .12(.36) | .18(.40) | .06 (.26) |
| Math matriculation | -.02(.27) | -.08(.33) | -.06(.32) |  | -.08(.37) | -.34 (.40) | -.26(.27) |
| Academic task values |  |  |  |  |  |  |  |
| Finnish | -.05(.19) | .15(.22) | .20(.15) |  | -.10(.22) | .08(.25) | .18(.16) |
| Math and Science | -.60(.17)*** | -.12(.19) | .58(.14)*** |  | -.89(.20)*** | -.25(.23) | .64(.15)*** |
| Humanities | .82(.21)*** | .32(.22) | -.41(.17)* |  | .62(.21)*** | .15(.21) | -.47(.17)* |
| Foreign language | .20(.16) | .02(.17) | -.18(.14) |  | .36(.20) | .02(.21) | -.34(.15)* |
| Practical subjects and arts | -.09(.16) | .02(.19) | .06(.14) |  | .09(.19) | .12(.19) | .03(.14) |
| Profiles |  |  |  |  |  |  |  |
| Vs. P1 (*Monetary-oriented*) |  |  |  |  |  |  |  |
| P2 (*Prospect-oriented*) | .27(.34) | .23(.38) | -.04(.32) |  | .17(.39) | .18(.46) | -.01 (.32) |
| P3 (*Family-oriented*) | .69(.33)* | 1.03(.39)* | .34(.35) |  | .65(.39) | 1.23(.46)* | .58(.41) |
| P4 (*Society-oriented*) | 1.39(.50)* | 1.81(.53)** | .42 (.41) |  | 1.53(.54)** | 2.12(.62)** | .59 (.46) |
| Vs. P2 (*Prospect-oriented*) |  |  |  |  |  |  |  |
| P3 (*Family-oriented*) | .42(.35) | .80(.36)* | .38(.32) |  | .48(.40) | 1.05(.40)* | .57(.46) |
| P4 (*Society-oriented*) | 1.12(.47)** | 1.58(.50)* | .46(.37) |  | 1.36(.51)* | 1.94(.55)* | .58(.42) |
| Vs. P4 (*Society-oriented*) |  |  |  |  |  |  |  |
| P3 (*Family-oriented*) | -.70(.37) | -.78(.41) | .08(.34) |  | -.88(.43)* | -.89(.44)* | -.01(.36) |

# External Appendix C: A Classified List of Majors/Professions for the Present Study

**Non-STEM occupations**

Leisure activities and youth work, administration, Construction and landscape workers, Agriculture, fishery, and forestry, Social services, Tourism, Hotel and catering, Domestic and consumer services, Cleaning services, Tourism, catering and domestic services, the Ministry of Education sector, Hairdressers, beauticians and related workers, Languages, History and archaeology ,Educational sciences ,Teaching and education (not math- or science-related fields), Journalists ,Crafts and design, Literature, Musicians, Creative and performing artists, Cultural and arts research, Sales, marketing and public relations professionals, Philosophy, Lawyer, Military and border guard, Fire and rescue services, Police services (Policy and planning management, police officers, policy administration professionals), government officials, Industrial management (including science-related managerial jobs)

**Support-level life science occupations**

Health associate professionals, Nurse, Biological engineering technicians, Dental assistants and therapists, Pharmaceutical technicians and assistants, Veterinary technicians and assistants

**Profession-level life science occupations**

Doctors, Dentists, Dieticians and nutritionists, Biological engineers, Biological science, Medical science, Veterinarians

**Support-level math-intensive occupations**

Industrial machinery mechanics and repairers, Broadcasting, telecommunications and Web technicians, Chemical and physical science technicians, Computer network and systems technicians, Electronics engineering technicians, mechanics and servicers, Financial and mathematical associate professionals

**Professional-level math-intensive occupations**

Mathematicians, actuaries and statisticians, Software, Web and multimedia developers, Computer network professionals, Physical and chemical professionals, Mechanical, metal and energy engineering, Electrical and automation engineering, Information and telecommunications technology, Graphics and communications technology, Food sciences, food industry and biotechnology, Automotive and transport engineering, secondary school teachers in math and science.

# External Appendix D: Demographic Factors and Matriculation Scores

**Demographic factors**

Gender and family socioeconomic status (SES) of the participants were included in our analyses. Gender was coded as 0 (female) or 1 (male). Parent occupational status was indicated by parents' occupations reported at grade 11. Each parent's occupation was first coded according to the Classification of socio-economic groups issued by Statistics Finland (1989). This measure was further recoded as 1 (unsalaried position), 2 (blue collar), 3 (lower white collar) and 4 (upper white collar). If both parents were working, the higher occupational status of either parent would be used as the indicator of the parent occupational status.

**Matriculation results in Finnish and math**

In the present study, matriculation examination results in Finnish and math were used because mother tongue is compulsory for everyone and about 80% of the candidates choose math as one of the other compulsory tests (Finnish Matriculation Examination statistics, 2007). Furthermore, as we were interested in STEM majors and careers, we controlled for the effect of math achievement in the models. The tests are graded according to normal distribution into seven categories. A rough literal translation of the grades and corresponding points is 0 = "not approved", 1 = "approved", 2 = "gladly approved", 3 = "approved with praise", 4 = "approved with much praise", 5 = "approved with exceptional praise", and 6 = "praised"/"lauded". By including this control we are isolating the predictive effect of academic and work values to that portion of variance unrelated to intra-individual variations in actual competencies across subject areas. It is important to note that this decision is very conservative because we know that actual competencies are major developmental predictors of academic task values.

# External Appendix E: Descriptive Statistics of the Sample and Tests of the Difference between Female and Male

Table E1

*Descriptive Statistics of the Sample and Tests of the Difference between Female and Male (N = 1,259)*

|  | Female (n=745) | Male(n=514) | Mean Test between-  gender difference |
| --- | --- | --- | --- |
| Academic STVs | Mean (SE) | Mean (SE) | T-Test |
| Finnish | 5.36(.03) | 4.69(.05)_c_ | 134.00*** |
| Math and Science | 4.52(.03) | 4.93(.04)_b_ | 30.59*** |
| Humanistic and social subjects | 5.15(.05)_a_ | 4.72(.06)_c_ | 24.24*** |
| Foreign language | 6.06(.04) | 5.61(.06) | 90.90*** |
| Practical subjects and arts | 5.12(.04)_a_ | 4.69(.06)_c_ | 35.53*** |
| Mean test of work value within gender | F = 153.5*** | F = 52.83*** |  |
| Personal work values | Mean (SE) | Mean (SE) |  |
| Monetary value | 4.67(.04)_d_ | 5.11(.04)_f,h_ | 45.03*** |
| Prospect value (i.e, Careerism) | 4.66(.04)_d_ | 4.93(.04)_g_ | 18.86*** |
| Society value (i.e., Altruism) | 4.13(.04) | 3.95(.05) | 90.07** |
| Family Values | 5.16(.04)_e_ | 5.02(.04)_f,g_ | 6.48* |
| People-orientation | 5.29(.03)_e_ | 5.24(.03)_h_ | 1.01 |
| Mean test of work value within gender | F = 153.5*** | F = 137.5*** |  |
| Control variables | Mean (SE) | Mean (SE) |  |
| Finnish matriculation | 4.59 | 4.24 | 13.91*** |
| Math matriculation | 4.14 | 4.55 | 16.13*** |
| SES | 3.29 | 3.28 | 0.02 |
| STEM majors or careers at age 25-27: | %^1^ | %^2^ | Chi-square Test |
| Non-STEM | 65 | 43 | 172.71*** |
| HBMS (professional- and support-levels) | 30 | 17 |  |
| MPECS (professional- and support-levels) | 5 | 40 |  |
| Frequency Test within gender | 306.98*** | 68.83*** |  |
| only professional-level | %^1^ | %^2^ | 41.59*** |
| HBMS (%) | 7 | 3 |  |
| MPECS (%) | 4 | 23 |  |

*Note.* ^1^ the percentage of females; ^2^ the percentage of males. Means within a column sharing the same subscripts (a-h) are not significantly different at the *p* < .05 level for the test of work value within gender. *** *p* <. 001; ** *p* <. 01; * *p* <. 05

# External Appendix F: Class Enumeration Process

*Figure F*. Elbow Plot of the Fit Indices of the Latent Profile Analyses

# External Appendix G: Relations between Work Value Profiles and Subject-Specific Task Value

Mean difference comparisons of academic STVs within and between each work value profiles are presented in Table G1 (see Table G2 for the similar results controlling for other factors). The levels of academic STVs in the five domains were significant within each profile. The *Monetary-oriented* group placed relatively higher STVs on math and science than on Finnish, social subjects, and arts, whereas the reverse was true for the *Family-* and *Society-oriented* groups. The *Prospect-oriented* group placed slightly higher STVs on social subjects and Finnish than on math/science and arts. However, individuals in all profile groups placed the highest STV on foreign language.

With respect to between-profile comparisons, individuals in the *Society-oriented* group had the highest STVs for Finnish and arts, followed by members of the *Prospect-oriented* group; both *Society-* and *Prospect-oriented* groups placed higher and similar STVs on social subjects and foreign language than the other groups. However, the *Prospect-oriented* group also placed high STV on math and science, as was also true of the members of the *Monetary-oriented* group. The *Monetary-oriented* group placed the lowest STVs on Finnish and arts; and individuals in the *Family-oriented* group placed the lowest STVs on math and science.

The present study is among the first to explore the relationship between core personal work values and academic STVs. Individuals in Different work value profiles had different levels and rank orderings of academic STVs. The *Society-* and *Family-oriented* groups placed relatively higher STVs on humanities and arts than on math and science, suggesting that people may perceive that humanities- and arts-related jobs allow them to more directly benefit society and put more time into family. Unsurprisingly, the *Money-oriented* group placed relatively higher values on math and science than on other subjects and groups, given that STEM majors dominate the list of top-earning college majors. The *Prospect-oriented* group tended to put high value on all subjects and may believe that balancing different subjects is potentially important for their career development and attainment. Interestingly, all profile groups placed the highest STVs on foreign language, suggesting that Finnish students believe that foreign language skills increase their educational and career opportunities

Table G1

*Mean Difference Comparisons within and between Each Profile*

|  | P1  (Monetary-oriented)  N = 295 |  | P2  (Prospect oriented)  N = 329 |  | P3  (Family-oriented)  N = 355 |  | P4  (society-oriented)  N = 280 | Mean test  between profiles |
| --- | --- | --- | --- | --- | --- | --- | --- | --- |
|  | Mean(SE) |  | Mean(SE) |  | Mean(SE) |  | Mean(SE) | F(3, 1259) |
| Task value |  |  |  |  |  |  |  |  |
| Finnish | 4.44(.06)_a_ |  | 5.31(.05)_b_ |  | 4.99(.06)_d_ |  | 5.63(.05)_e_ | 79.55*** |
| Math and science | 4.99(.05) |  | 4.99(.04)_c_ |  | 4.37(.06) |  | 4.41(.04) | 21.55*** |
| Humanistic and social subjects | 4.54(.07)_a_ |  | 5.44(.07)_b_ |  | 4.72(.08)_d_ |  | 5.51(.08)_e_ | 70.45*** |
| Foreign language | 5.75(.06) |  | 6.14(.05) |  | 5.59(.07) |  | 6.08(.05) | 33.5*** |
| Practical subjects and arts | 4.42(.07)_a_ |  | 5.04(.06)_c_ |  | 4.83(.07)_d_ |  | 5.54(.06)_e_ | 44.53*** |
| Mean test of STVs  within each profile | F = 80.69*** |  | F = 69.25*** |  | F = 46.98*** |  | F = 119.9*** |  |
| Parent occupational status | 3.25(.05) |  | 3.27(.04) |  | 3.30(.04) |  | 3.35(.04) | 1.03 |
| Matriculation |  |  |  |  |  |  |  |  |
| Finnish | 4.27(.19) |  | 4.42(.15) |  | 4.63(.18) |  | 4.39(.19) | .47 |
| Math | 4.62(.16) |  | 4.75(.17) |  | 4.15(.18) |  | 4.07(.17) | 13.91*** |

*Note.* Means within a column sharing the same subscripts (a-e) are not significantly different at the p < .05 level based on the mean test of STVs within each profile; f the percentage of females; g the percentage of males. *** p <. 001.

Table G2

*Mean difference comparisons within and between each profile controlling for gender, matriculation, and parent occupational status*

|  | P1  (Monetary-oriented) |  | P2  (Prospect oriented) |  | P3  (Family-oriented) |  | P4  (society-oriented) | Mean test  between profiles |
| --- | --- | --- | --- | --- | --- | --- | --- | --- |
|  | Mean(SE) |  | Mean(SE) |  | Mean(SE) |  | Mean(SE) | F(3, 1259) |
| Mean |  |  |  |  |  |  |  |  |
| Finnish | 4.46(.10)_a_ |  | 5.32(.08)_b_ |  | 4.99(.10)_d_ |  | 5.62(.09)_e_ | 79.55*** |
| Math and science | 5.01(.10) |  | 5.04 (.09)_c_ |  | 4.37(.10) |  | 4.44(.09) | 21.55*** |
| Humanistic and social subjects | 4.54(.10)_a_ |  | 5.44(.09)_b_ |  | 4.71(.10)_d_ |  | 5.49(.09)_e_ | 70.45*** |
| Foreign language | 5.75(.10) |  | 6.13(.09) |  | 5.65(.10) |  | 6.05(.09) | 33.5*** |
| Practical subjects and arts | 4.51(.07)_a_ |  | 4.97(.06)_c_ |  | 4.81(.10)_d_ |  | 5.44(.06)_e_ | 44.53*** |
| Mean test of STVs  within each profile | F = 59.15*** |  | F = 53.90*** |  | F = 43.71*** |  | F = 85.28*** |  |

*Note.* Means within a column sharing the same subscripts (a-e) are not significantly different at the *p* < .05 level based on the mean test of STVs within each profile.
